# A retrospective study of trauma-associated oral and maxillofacial lesions in a population from southern Taiwan

**DOI:** 10.1590/S1678-77572010000100003

**Published:** 2010

**Authors:** Jing-Yi CHEN, Wen-Chen WANG, Yuk-Kwan CHEN, Li-Min LIN

**Affiliations:** 1 DDS, Chief Resident, Department of Oral Pathology, School of Dentistry, Kaohsiung Medical University Hospital, Kaohsiung, Taiwan.; 2 DDS, MS, Assistant professor, Department of Oral Pathology, Faculty of Dentistry, College of Dental Medicine, Kaohsiung Medical University & Hospital, Kaohsiung, Taiwan.; 3 DDS, MS, Assistant professor, Head of Department of Oral Pathology, Faculty of Dentistry, College of Dental Medicine, Kaohsiung Medical University & Hospital, Kaohsiung, Taiwan.; 4 DDS, MS, PhD, Professor, Department of Oral Pathology, Faculty of Dentistry, College of Dental Medicine, Kaohsiung Medical University & Hospital, Kaohsiung, Taiwan.

**Keywords:** Trauma, Soft tissue, Hard tissue, Biopsy

## Abstract

**Objective:**

The aim of this retrospective analysis was to determine the age, gender, frequency and distribution of trauma-associated hard tissue and soft tissue lesions of the oral and maxillofacial region in a population from southern Taiwan.

**Patients and Methods:**

Approximately 10% of the 27,995 biopsy records of patients with history of trauma resulting in lesions who were treated at our institution between 1991 and 2006 were examined for this study.

**Results:**

In the included records, there were 2,762 soft tissue and 26 hard tissue lesions. Mucocele was the most frequent trauma-associated soft tissue lesion (955 cases). The youngest patients were those who presented with mucocele (mean age = 27.3 years), while the oldest patients were those with peripheral giant cell granuloma (58 years). The lower lip was the most frequent site of occurrence of mucocele (676, 64.5%) and was also the predominant site of occurrence of all soft tissue lesions (815, 29.5%), followed by the buccal mucosa (654, 23.4%) and the tongue (392, 14.2%). Trauma-associated hard tissue lesions included only osteoradionecrosis (24 cases) and traumatic bone cysts (2 cases).

**Conclusion:**

As little data of this nature have been reported from populations of Asian developing countries, the findings of this retrospective analysis is valuable for epidemiological documentation of type of traumatic oral lesions as well as for informing the professionals and the layman about the importance of this category of oral lesions.

## INTRODUCTION

Trauma can be divided into mechanical, thermal and chemical injuries. Injuries sustained during the course of routine daily activities or in motor vehicle accidents accounts for the large majority of trauma-associated lesions occurring in the oral and maxillofacial region^5,16^. On the other hand, tooth attrition can be seen in those who chew betel-quid or suffer from bruxism^3^. Tooth cervical abrasion may also be a consequence of vigorous tooth brushing^3^. Even dental or medical treatment can cause injuries to oral mucosa and teeth, such as radiation-induced mucositis, radiation caries and osteoradionecrosis^3,6^. Other causes of oral and maxillofacial injury include self-inflicted physical injuries (voluntary/involuntary habits and factitious injury), thermal and chemical burns^16^. Furthermore, oral piercing to tongue/lip is another cause of oral trauma^13^. Therefore, in summary, oral and maxillofacial trauma can be categorized with respect to a variety of factors including etiology, anatomy, pathology and therapeutic considerations as well as occasional (single-event injury) and habit-related (repeated injury)^1,16,17^.

The review of the English-language literature, show that studies of trauma-associated lesions of the oral and maxillofacial area in populations of Asian developing countries are relatively uncommon, and those that do exist are mostly limited to studies of jawbone fractures^4,9^ or traumatic injuries to teeth^2^. In addition, there is a high frequency of betel-quid chewers and patients with oral malignancies receiving radiation treatment as well as a high frequency of motorcyclists amongst the population of southern Taiwan. Thus, it is useful to analyze data on this subject. Archival biopsy records of the oral and maxillofacial region within a population of patients from southern Taiwan were reviewed in the present study relative to age, gender, frequency and distribution.

## PATIENTS AND METHODS

The Oral Pathology Department of Kaohsiung Medical University Teaching Hospital is the only department providing the biopsy service of oral and maxillofacial region for southern Taiwan, receiving specimens mainly from the surgeons of the Departments of Oral & Maxillofacial Surgery, Ear, Nose & Throat and Plastic Surgery of our hospital as well as other nearby regional hospitals and local dental clinics. The three experienced board-certified oral and maxillofacial pathologists of our Oral Pathology Department make the histological diagnosis for each biopsy independently, based mainly on paraffin-embedded sections of hematoxylin-eosin staining and sometimes conjunction with immunohistochemical staining and histochemical staining. All histological diagnoses are confirmed by peer slide review. However, if disagreement exists amongst the pathologists, a consensus is reached upon discussion.

Biopsy records of 27,995 microscopically diagnosed cases of lesions in the oral and maxillofacial region (1991-2006) were retrieved from our department files. Among these biopsy records, those with histological diagnosis related to an etiology of trauma according to the literature were identified^14^. A total of 2,938 cases of trauma-associated soft tissue or hard tissue lesions were selected. An additional inclusion criterion was that there must have been history of trauma to the head and neck region recorded by the clinicians. A total of 150 cases were then excluded for analysis due to a lack of trauma history. Finally, a pool of 2,788 cases of trauma-associated soft tissue or hard tissue lesions with pre-existing trauma history was selected for detailed analysis (Tables 1 and 2). Information regarding age, gender, location and histologic diagnosis was compiled from the patients' medical charts for each case.

**Table 1:** Demographic data of the trauma-associated hard tissue lesions (26 cases)

**Lesions**	**Cases N(%)**	**M:F ratio**	**Mean age, range (yrs)**	**Sites (number, percentage)**
Osteoradionecrosis*	24 (92.3%)	11:1	57.4, 39-83	Mandible (19,79.2%)
Maxilla (5, 20.8%)				
Traumatic bone cyst**	2 (7.7%)	0:1	22.0, 12-32	Mandible (2, 100.0%)

M: Male; F: Female.

* Eight cases (33.3%) were initiated by tooth extraction, 7 cases (29.2%) by periodontitis, and 4 cases (16.7%) by apical lesions, 5 cases (20.8%) were spontaneous; radiation therapy dose ranged from 4,600 to 7,380 cGy.

** Both cases had motorcycle-related injuries.

**Table 2:** Demographic data of the trauma-associated soft tissue lesions (2762 cases)

**Lesions**	**Cases N(%)**	**M:F ratio**	**Mean age, range (yrs)**	**Three most common sites (number, percentage)**
**Mucocele**	955 (34.5%)	1.4:1	27.3, 1-74	Lower lip (676, 64.5%) Floor of the mouth (100, 10.5%) Buccal mucosa (93, 9.7%)
**Traumatic ulcer**	818 (29.6%)	2.7:1	51.7, 8-83	Buccal mucosa (344, 42.0%) Tongue (201, 24.6%) Lower lip (73, 8.9%)
**Irritation fibroma**	701 (25.4%)	0.8:1	43.8, 3-96	Buccal mucosa (200, 28.5%) Tongue (116, 16.6%) Lower lip (58, 8.3%)
**Inflammatory fibrous hyperplasia/epulis fissuratum**	258 (9.3%)	1.1:1	56.6, 11-88	Gingiva (92, 35.7%) Buccal mucosa (30, 11.6%) Vestibule (20, 7.8%)
**Hematoma**	24 (0.9%)	2.0:1	44.0, 9-74	Tongue (7, 29.2%) Buccal mucosa (4, 16.7%) Lower lip (4, 16.7%)
**Traumatic neuroma**	4 (0.2%)	3.0:1	51.3, 16-67	Vestibule, neck, preauricular, alveolar mucosa (1 of each, 25%)
**Peripheral giant cell granuloma**	2 (0.1%)	1.0:0	58.0, 54-62	Gingiva (2, 100%)

M: Male; F: Female.

## RESULTS

A total of 2,788 oral biopsies performed between 1991 and 2006 (approximately 10% of all specimens existing our Department's files) with a confirmed history of trauma related to head neck area recorded by the clinicians were classified as trauma-associated soft tissue or hard tissue lesions. The majority of the cases (2,762 specimens) were soft tissue lesions; there were only 26 cases of hard tissue lesion (osteoradionecrosis, 24 cases; traumatic bone cyst, 2 cases) (Table 1). The majority of the patients with osteoradionecrosis were male and the lesions occurred in the mandible with radiation therapy history (4600 to 7380 cGy) (Table 1). Both cases of traumatic bone cyst occurred in the mandible of female patients having a history of motorcycle-related injuries to head neck area (Table 1).

The demographic data of the patients with trauma-associated soft tissue lesions are summarized in Table 2. Mucocele was the most common lesion (955 cases, 34.5%), followed by traumatic ulcer (818 cases, 29.6%), irritation fibroma (701 cases, 25.4%) and inflammatory fibrous hyperplasia/epulis fissuratum (258 cases, 9.3%); the first three cases contributed nearly 90% of all the traumatic-associated soft tissue lesions. A wide age range was noted for all types of soft tissue lesions, with the exception of peripheral giant cell granuloma. Patients with mucocele were the youngest (mean age = 27.3 years), whilst the oldest patients were those with peripheral giant cell granuloma (mean age = 58 years). The lower lip was not only the most frequent site of occurrence of mucocele (676, 64.5%) but was also the predominant site of occurrence of all other trauma-associated soft tissue lesions (815, 29.5%). The second most common sites of occurrence were the buccal mucosa (654, 23.4 %) and the tongue (392, 14.2%).

Most of the histological diagnoses of the present series of patients were consistent to the clinical impressions of the surgeons with the exception of the two cases of traumatic bone cyst (clinical diagnosis as odontogenic cyst, benign odontogenic tumor respectively), 35 cases of mucocele, 4 cases of traumatic neuroma and 2 cases of peripheral giant cell granuloma with most initial clinical impressions as irritation fibromas.

## DISCUSSION

The Oral Pathology Department of our hospital not only provides services for almost all biopsied cases of oral and maxillofacial lesions (Medical Pathology contributes only with a very small number of cases), but is also the most heavily used referral center for patients with these lesions in southern Taiwan. Therefore, the various types of trauma-associated lesions in this study are representative of the occurrence of such lesions in this geographical region.

The top three trauma-associated soft tissue lesions (mucocele, traumatic ulcer and irritation fibroma) have accounted for nearly 90% of the present biopsy-based cohort, in which some details of the traumatic insult could not be precisely identified. Nevertheless, accidental biting, trauma by hand-held implements (e.g. toothpick, pencil, tooth brush), and other traumatic oral habits, such as lip biting or sucking would be among the major causes of these three soft tissue traumatic lesions. On the other hand, the most frequent (about 90%) trauma-associated hard tissue lesion was osteoradionecrosis in which 8 cases (25.8%) were initiated by surgical insult of tooth extraction.

Oral ulcers with possible traumatic etiology are not usually subjected to biopsy. The frequencies of traumatic ulcer of the present study may be overestimated because these cases were selected on the basis of the histopathological diagnostic records, and some of these patients may have suffered tissue damage due to other specific ulcerative conditions (e.g., vesiculobullous diseases, erosive lichen planus, Bechet's disease) unrelated to trauma. The typical pathological findings of such specific ulcerative entities may not be accurately revealed upon histologic examination due to sampling errors. On the other hand, osteoradionecrosis is also usually not to have biopsy because further surgical intervention may worsen the disease. Between 1991 and 2006, only 24 osteoradionecrosis specimens were acquired from surgery in order to control the spread of the diseases, which were complicated with severe oral infection. Therefore, the small number of cases of osteoradionecrosis for the present biopsy-based cohort cannot reflect the true incidence of the disease.

Although several articles report on the frequency of oral lesions based on clinical or histologic information, trauma history and sites of occurrence were not confirmed in these studies^7,10-12,14^. In the present study, it was found that the type and site of trauma-associated lesions varied with age. Patients with mucocele were generally younger than patient groups exhibiting other types of traumatic soft tissue lesions. For example, irritation fibroma, inflammatory fibrous hyperplasia/epulis fissuratum and peripheral giant cell granuloma typically occurred in older patients. The latter two types of lesions occurred most commonly on the gingiva and were frequently related to ill-fitting prostheses, which is compatible with the results of the study by García-Pola Vallejo, et al^8^ (2002).

The number of trauma-associated hard tissue lesions confirmed by microscopic examination of biopsy specimens was much smaller than that of the soft tissue counterparts. This may be due to the fact that, without biopsy confirmation, radiographic evidence was by no means diagnostically definitive.

In conclusion, little data of this nature have been reported from populations of Asian developing countries. Thus, the information derived from the present study is valuable for epidemiological documentation of type of traumatic oral lesions as well as for informing professionals and laymen about the importance of this category of oral lesions. Finally, a survey of traumatic oral lesions derived from biopsy records, as demonstrated in this study, has the merit of inherent certainty of definitive diagnosis, which is not possible in studies based on clinical observation alone.
